# Mechanism of Protein-Z-Mediated Inhibition of Coagulation Factor Xa by Z-Protein-Dependent Inhibitor: A Molecular Dynamic Approach

**DOI:** 10.5402/2012/762728

**Published:** 2012-03-20

**Authors:** Mohammad Reza Dayer, Omid Ghayour, Mohammad Saaid Dayer

**Affiliations:** ^1^Department of Biology, Faculty of Science, Shahid Chamran University, Ahvaz 6198864936, Iran; ^2^Department of Mathematics, Faculty of Mathematical and Computer Sciences, Shahid Chamran University, Ahvaz, Iran; ^3^Department of Parasitology and Medical Entomology, Tarbiat Modares University, Tehran, Iran

## Abstract

Protein Z is a plasma protein functioning as a carrier for ZPI. Protein Z also accelerates inhibitory effect of ZPI on factor Xa by 1000-fold. Inhibition of coagulation cascade via FXa by ZPI and other serpins is very important safety factor for normal homeostasis protecting human life against unwanted thrombosis. In the present work using native structure of PZ, ZPI, FXa and in a dynamic simulation, using NAMD software, the ternary complex was studied in an up to 10 nanoseconds protocol. Rely on trajectory analyses, we postulated that PZ binds ZPI by using its SP-like domain and through noncovalent forces. PZ then transfers ZPI through-out the blood, and by using its GLA domain and a bivalent cation of calcium, PZ binds to phospholipid bilayers (e.g., platelet) where the FXa is preallocated. In case of PZ-ZPI binding to plasma membrane, a series of complementary interactions take place between FXa, and PZ-ZPI complex including interactions between RCL loop of ZPI and catalytic site of FXa and some take place between long arm of PZ (composed of GLA, EGF1, and EGF2 domains) and GLA domain of FXa. In our claim these complementary interactions lead PZ to bind correctly to prelocated FXa.

## 1. Introduction

Human protein Z, that is, PZ, is a single-chain plasma glycoprotein which has been synthesized in liver and endothelial cells with 62-KD and 360 residues [1]. PZ has plasma concentration of 2.9 ± 1.0 *μ*g/mL [2, 3]. In the primary structure of PZ, there are four distinct and consecutive domains, which from N- to C-terminus, respectively, are named GLA, EGF1, EGF2, and SP-like domains. GLA domains, which include residues 1–46, contains 13 gamma carboxy glutamic acid, GLA, which have been formed by vitamin-K-dependent posttranslational carboxylation of relevant glutamic acid residues. GLA domain also contains a single disulphide bridge between Cys18 and Cys23 [4–9]. GLA domain plays an important role in PZ physiological function. Negatively charged GLA domain using bivalent cation of Ca^+2^ helps PZ to bind to negative groups of phospholipid membranes, where PZ acts as a cofactor for FXa inhibition by ZPI. However PZ with low content of GLA lacks cofactor activity [4, 5, 10–12]. It was also shown that GLA domain accelerates PZ interaction with FXa on phospholipid bilayers by about 6 times faster [10, 13]. The next two domains in PZ are epidermal-growth-factor-like domains, EGF1 and EGF2. EGF1 is stretched from residue 47 to 83 and EGF2 is located between residue 85 and 126. Each of which contains 3 disulphide bonds. The last domain in PZ is serine-protease-like (SP-like) domain beginning from residue 135 and continuing to the last residue of PZ (C-terminal residue number 360). SP-like domain of PZ has two disulphide bonds which is thought to have an active site resembling that of the triad active site of serine protease family. Serine protease enzymes such as FVII and FIX in their triad active site contain His57, Asp162, and Ser195, which are essential for catalytic activity, but in contrast, in PZ His and Asp, have been replaced by Thr and Ala, respectively [8–10, 14–17]. Human ZPI is a serine protease inhibitor (serpin), synthesized predominantly in liver, with about 25–30% sequence homology to other serpins such as antithrombin III (AT3) [18–21]. ZPI makes a complex with PZ in plasma with dissociation constant of 7 nM [22]. PZ/ZPI complex is more effective than ZPI in inhibition of FXIa and FIXa [14, 18]. ZPI in its primary structure contains three beta sheets named A, B, and C, eight to nine alpha helices, hA,…,hI, and a reactive RCL loop which is composed of four residues, Thr385-Ser388, also known as P3-P1′ (P3-P2-P1-P1′) in the Scheeter-Berger nomenclature method. ZPI inhibits FXa through complementary binding of RCL loop and interaction with catalytic site of FXa resulting in ZPI-FXa complex formation [11, 23–26]. However ZPI-FXa complex is unstable because FXa proteolytically cleaves RCL loop of ZPI at P1 (Tyr387) site, and the products are released from FXa active site, [13, 27]. In addition to the stereospecific interaction between RCL loop of ZPI and FXa active site, the specificity of FXa inhibition by ZPI is guaranteed by additional exosite interactions between ZPI body and FXa surface residues. In this context there are several electrostatic interactions between positively charged groups of autolysis loop (residues 143–154 and its proximity) of FXa including Lys96, Arg143, and Lys147 with negatively charged groups on complementary region on ZPI including Glu313, Glu231, and Asp233, respectively [24, 28]. Since FXa inhibition by ZPI in the presence of PZ is increased 1000 fold and there are miscellaneous reports showing that PZ itself interact with FXa, then it had been postulated that PZ may have different function in FXa inhibition by ZPI:

1. making stable complex with ZPI and act as plasma carrier for ZPI,
2. helping ZPI to immobilize on phospholipid membrane where the FXa is flanked,
3. participating in targeting and docking of ZPI on FXa, to aid the stereospecific interactions between ZPI and FXa [10, 13, 22, 27, 28].

The precise mechanism of PZ-mediated promotion of FXa by ZPI and also the molecular-/atomic-level events of this phenomenon are not well understood. The main scope of the present work is to mechanistically survey the successive interaction of PZ, ZPI, and FXa, using molecular dynamical approach by carrying out different simulations. We decided to study the time course event in hope to approve the postulated mechanism and to extract more details of molecular interaction.

## 2. Method and Material

### 2.1. Coordination Files Sources and System Setup

Optimized full-length PZ coordination files including GLA domain and calcium were obtained as a gift from Professor Pedersen [29]. ZPI and FXa files were obtained from protein data bank with PDB ID 3H5C and 2XBY, respectively [30, 31].

### 2.2. Complex Preparation

Binary complex of PZ-ZPI was constructed by superimposing residue 1–86 of full-length PZ protein to same residues in protein 3H5C which consist of PZ-ZPI complex lacking GLA of PZ by VMD software [32]. Ternary complex of PZ-ZPI-FXa was constructed by docking optimized structure of FXa with PDB ID of 2XBY to 10 nanoseconds simulated binary complex of PZ-ZPI. By using HEX 6.3 software, we docked FXa to ZPI in such a way to put RCL loop of ZPI inside FXa catalytic site. Also the best positions with lowest energy have been chosen for further studies [33], and trajectory analyses were done by using VMD and Vega ZZ softwares [32–34]. 

### 2.3. Simulation Conditions

Proteins were placed in cubic boxes with dimensions large enough to equilibrate the protein within them, and the protein did not exit the box during simulation. The box size of 63.17 × 95.22 × 111.34 Å is used for PZ, box size of 66.25 × 89.36 × 66.93 Å for ZPI, and box sizes of 58.80 × 72.54 × 65.15 Å for FXa. The box size of 118.35 × 136.27 × 131.36 Å and 127.02 × 127.86 × 120.84 Å were used for PZ-ZPI and PZ-ZPI-FXa complexes, respectively. TIP3P water was used as aqueous medium [35]. All the systems were energy minimized for up to 10000 minimization steps in order to remove bad contact in protein structure. Na^+^ and Cl^−^ ions were used as counterions for neutralizing the net charges of proteins. To study the effect of calcium ions, we simulate the entire system containing PZ protein with its GLA domain in the absence and the presence of 11 calcium ions. All the simulations were done in NPT conditions for 310 K of temperature and 1.0 atm. of pressure, and Particle mesh Ewald (PME) algorithm was applied to calculate electrostatic forces in our systems. The cutoff of 12 angstrom and time step of 2 fs were used in all simulations [36].

Molecular dynamic calculation of single proteins and their complex in solution was performed using NAMD 2.7, as a parallel code designed for high-performance simulation of large biological macromolecules, using CHARMM27 force filed [36, 37].

## 3. Results and Discussion

**Figure 1:**
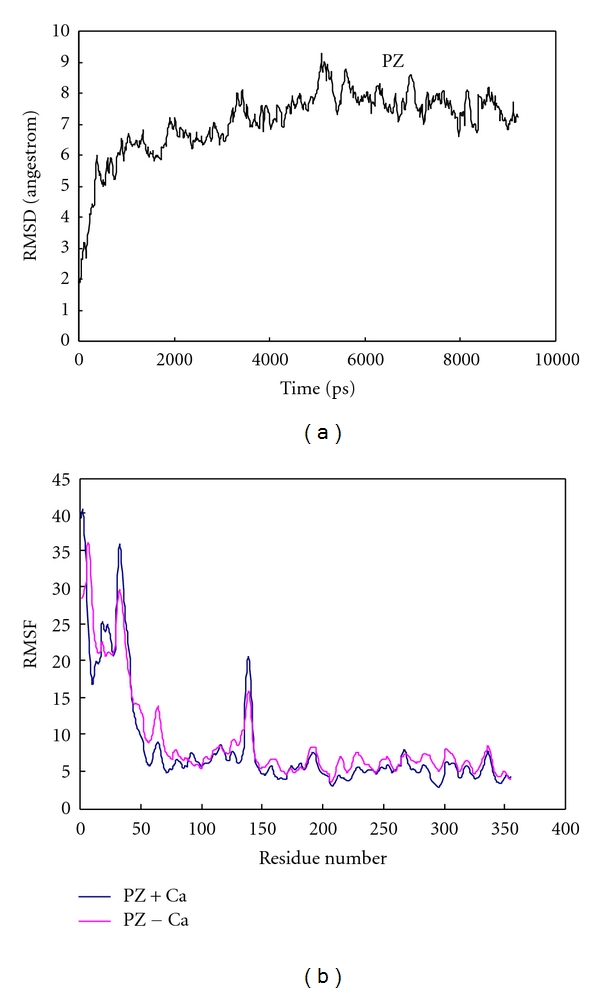
(a) Root mean square displacement (RMSD) of backbone to backbone for free PZ protein in aqueous solution at 310 K and 1 atmosphere of pressure. (b) Root mean square fluctuation (RMSF) for each alpha carbons of PZ in the presence (blue) and absence (red) of calcium ions.

In order to study the mechanism by which PZ accelerates ZPI to inhibit FXa, we simulate PZ, ZPI, FXa, PZ-ZPI binary complex, and PZ-ZPI-FXa ternary complex in separated simulation.  Figure 1(a) shows the changes in root mean square displacement (RMSD) of PZ during simulation for up to 10 ns.

RMSD change of PZ confirms system stability during simulation. There are continuous small jumps in RMSD appearing each 2 ns of simulation and followed by a new stability state. Trajectory movie shows that these jumps in RMSD is caused by the bending of the long arm of PZ which contains EGF1, EGF2, and GLA domains. The bending movement seems to be restricted in some cases: (1) in case of binding of calcium to GLA domain, (2) in the case of binary complex formation between PZ and ZPI, and (3) in the case of ternary complex formation with ZPI and FXa.  Figure 1(b) shows the root mean square fluctuation, or RMSF, of each atom during simulation for PZ in the presence and in the absence of calcium ions. There are two hot regions with high fluctuating RMSF. The first region belongs to ~45 residues of N-terminal residues which move freely during simulation.

The second region, which appears as a sharp band at residues 135–146, is at the beginning of SP-like domain. This region is acting as flexible hinge forming a bending arm for PZ [38]. When calcium ions bind to GLA domain or when PZ makes binary or ternary complexes with ZPI or ZPI-FXa, respectively, the band altitude decreased prominently. We do hypothesize that this flexible arm may help PZ to match better counterproteins in complex formation. It is reasonable to expect that the association of two complementary proteins reduces the RMSF of their hot points.

**Figure 2:**
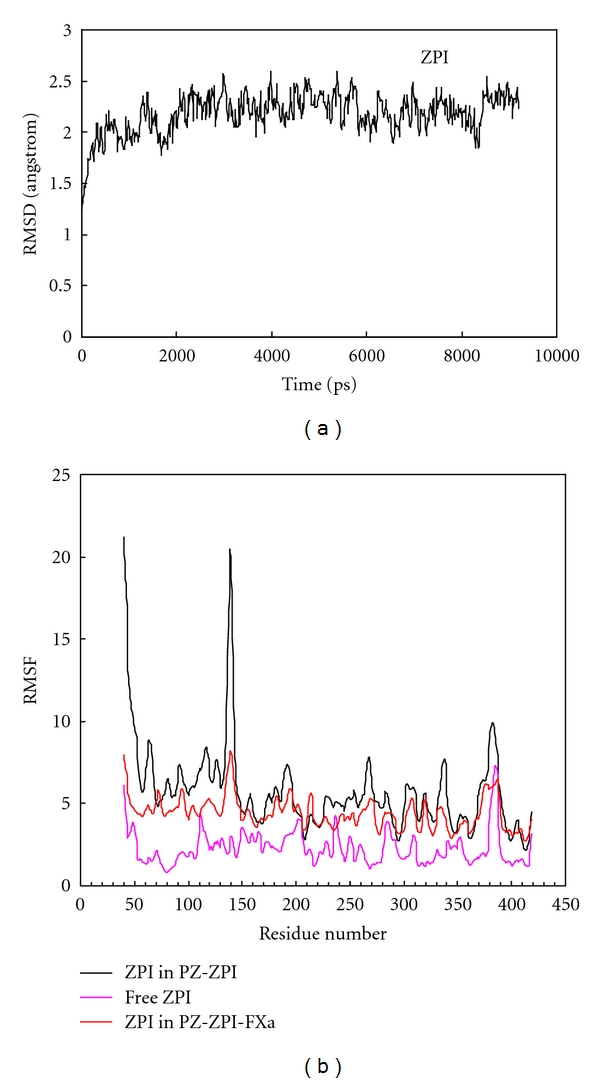
(a) Root mean square displacement (RMSD) of free Z-dependent protease inhibitor (ZPI) obtained in aqueous solution at 310 K and 1 atmosphere of pressure and a total time of 10 ns. (b) Root mean square fluctuation (RMSF) of all residues of free ZPI (violet), ZPI in PZ-ZPI complex (black) and in ternary complex of PZ-ZPI-FXa (orange) at 10 ns of simulation.

Figure 2(a) shows the RMSD of free ZPI. RMSD plot for ZPI for up to 10 nanoseconds indicates the stability state without any unwanted perturbations.  Figure 2(b) shows RMSF of ZPI complex with PZ and to PZ-FXa. RMSF changes of ZPI introduce two flexible points in ZPI structure, as shown. The first flexible point placed at the proximity of residue 139 and is the second at reactive center loop (RCL) of ZPI around residue 384 [39].  Figure 2(b) also shows that PZ binding to ZPI increases the flexibility of these two points. Accordingly it could be concluded that upon PZ binding to ZPI, the increased flexibility of these two points facilitates ZPI binding to FXa. The binding of ZPI to FXa, as is expected, decreases RMSF fluctuation especially in RCL loop, the FXa catalytic binding site.

**Figure 3:**
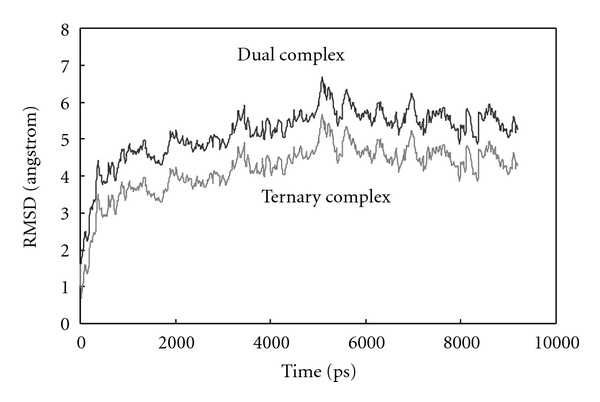
Root mean square displacement (RMSD) of binary complex of PZ-ZPI and ternary complex of PZ-ZPI-FX for up to 10 ns in aqueous solution at 310 K and 1 atmosphere of pressure.

The backbone RMSD changes of binary and ternary complexes during simulation in the presence of calcium ions are shown in Figure 3. RMSD changes for ternary complex show more stability of the complex system in comparison to binary complex. More stability of ternary complex confirms the effectiveness of ternary complex or inhibitory complex of PZ-ZPI-FXa formation in contrast to binary complex.

**Figure 4:**
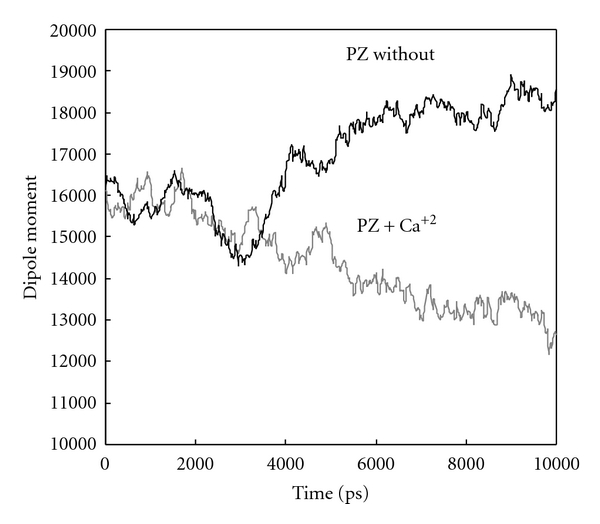
Dipolmoment changes of PZ in the presence (cyan) and absence (black) of calcium ions during simulation for up to 10 ns.

Figure 4 shows the PZ dipole moment changes in the presence and absence of calcium ions. The curve shows that at the presence of calcium ions the dipolmoment of PZ decreased significantly. GLA domain has 13 GLA residues each with two negative charges and makes a strong dipole moment with negative head arranged toward membrane phospholipids which builds up huge repulsive force preventing GLA binding to phospholipids bilayer. Binding of calcium to GLA decreases its dipole moment and even converts this repulsive force to an attractive force thereby facilitates PZ binding to phospholipids bilayer through GLA domains. This finding is in complete agreement with Rezaie experimental findings who had reported that PZ participate in ZPI targeting to phospholipids membranes where the FXa is flanked. In this way PZ increases the local concentration of ZPI 1000-fold [13, 28, 40].

**Figure 5:**
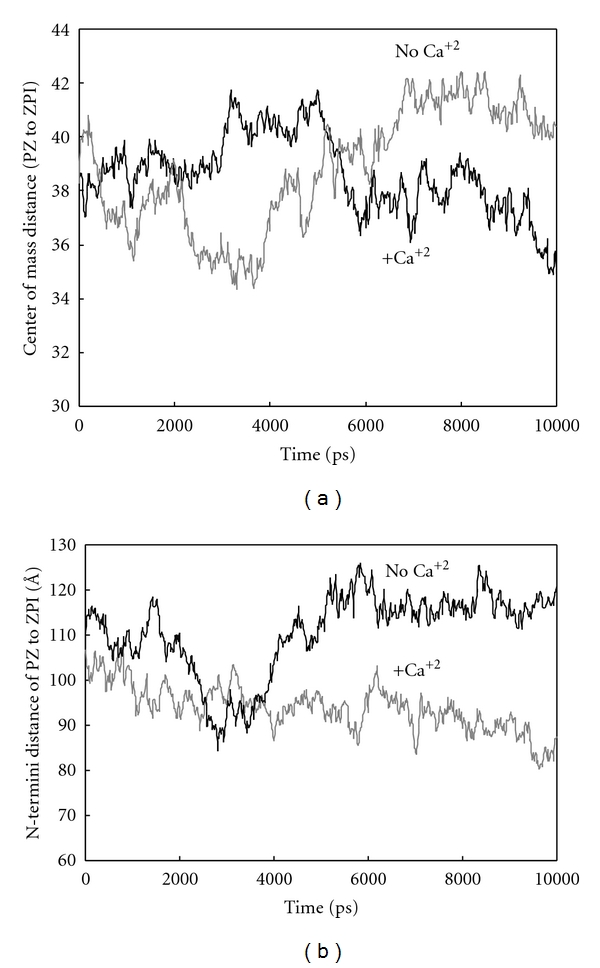
(a) Changes in the distance between center of mass of PZ and ZPI in binary complex in the presence and absence of calcium ions during simulation. (b) Changes in the distance between two N-termini of PZ and ZPI during simulation in the presence and absence of calcium ions.

Figure 5(a) shows the changes in distance between center of mass of PZ and ZPI in PZ-ZPI complex during simulation in the presence and absence of calcium ions. The curve shows that in the presence of calcium ions, and after 6 ns of simulation, the distance is decreased and the two proteins become attracted to each other.  Figure 5(b) illustrates the changes in distance between two N-termini of PZ and ZPI during simulation in the presence and absence of calcium ions. As it could be seen, this distance decreased significantly after 4 ns and the two N-termini of PZ and ZPI are pulled to each other along PZ axis. Figures 5(a) and 5(b) reveal that the presence of calcium ion causes ZPI attraction toward PZ center of mass and sliding down PZ axis, to proximity of GLA domain and to the place where FXa is immobilized on phospholipids membrane. This is facilitating ternary complex formation with FXa, an inhibitory complex. It had been shown that PZ not only participates in ZPI targeting toward phospholipids membrane but also activates ZPI with RCL loop pushed out from beta sheet and become suitable for entrance into catalytic site of FXa [30]. Our findings show that the distance between Ser388, a residue from RCL, to Asp102 (hydrogen-binding counterresidue), a residue from FXa, is decreased during simulation, and these two residues become in close proximity and imply that PZ binding affects RCL loop entrance to FXa active site [39, 40]. It had been reported that mutation in Arg255 of PZ inactivates ZPI function. Pederson relied on these reports and using molecular dynamic simulation and wild-type and mutated PZ (Arg255→His255), postulated that Arg255 in PZ is hydrogen bonded to Arg319 and Glu320 of PZ, so its replacement with His weakens this hydrogen binding and disturbs inhibitory effect of ZPI on FXa [29]. Our simulation data are presented in Table 1.

Though the distance between alpha carbons of Arg255 and Arg319 and Glu320 before and after simulation is lesser than that between other neighboring residues, the distance between potential groups for hydrogen bond formation is not necessarily the same. Our data show that functional groups of Glu92 are more accessible for hydrogen binding because of their closer vicinity to Arg255, 1.68 Å against 3.27 Å. The last frame of 100 nanoseconds trajectory shows that this stable hydrogen bond is very important for the final conformation of ternary complex and is placed in critical point of PZ arm near the flexible hinge. This hydrogen bond is also participating in PZ conformation and reduction of RMSF fluctuation.

**Figure 6:**
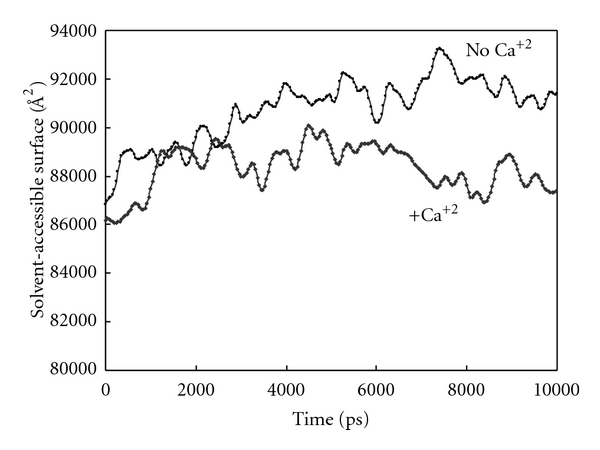
Changes in hydrophobic solvent-accessible surface (SAS) of the ternary complex of PZ-ZPI-FXa in the presence and absence of calcium for up to 10 ns of simulation in aqueous solution at 310 K and 1 atmosphere of pressure.

Figure 6 shows the changes in hydrophobic solvent-accessible surface (SAS) of ternary complex during simulation in the presence and absence of calcium ions. Decrease in hydrophobic SAS, which implies the increase in hydrophilic SAS of ternary complex of PZ-ZPI-FXa, indicates that in the presence of calcium ions the complex changed to functional complex with more hydrophilic interaction, for example, more hydrogen bonds and more hydration of proteins charged groups by surrounding solvent. These data indicate the formation of more folded, more hydrophilic ternary complex during simulation. It had been postulated that the efficiency of complex formation between FXa and ZPI is dependent first on the specific interactions of RCL loop with the active site of FXa and second on the complimentary interactions between residues lying in the outer surface of FXa body with counterpart groups on ZPI molecule. The interactions of RCL loop and FXa active site act as recognition tools for complex formation. P1 tyrosine (Tyr387) enters the S1 active site of FXa first then followed by the insertion of RCL loop to active site, and afterward miscellaneous interaction will be formed between P1–P3′ residues of ZPI and FXa active site residues. Rezaie had shown that preincubation of PZ protein with calcium ions promotes the rate of FXa inhibition by ZPI at least 5-fold of magnitude. Rezaie also shows that PZ acts only on membrane-bound FXa, and membrane binding of PZ is the rate-limiting step in PZ-ZPI-FXa ternary complex formation [13, 30, 39, 40]. In globular proteins, Rg or radius of gyration, is used as a measure of molecular radius.

**Figure 7:**
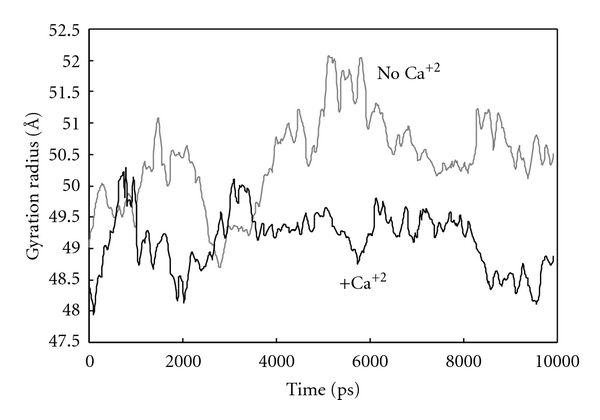
Changes in gyration radius (Rg) of the ternary complex of PZ-ZPI-FXa in the presence and absence of calcium ions.

Figure 7 exhibits the Rg variation of ternary complex during simulation. As it could be expected, there is a significant time course decrease in Rg on complex when it reaches the stable state. This decrement in Rg is in well agreement with our findings regarding formation of a more compacted, more hydrophilic ternary complex in simulation. In this context, following up the changes in hydrogen bonds during simulation gives us extra confirmatory documents. Our results show that running molecular dynamic simulation itself causes the ternary complex to feel or sensate the surrounding aqueous solution in such a way that their functional groups changed and corrected their orientation against water molecules and their nonbonded interactions. Hydrogen bonds are one of the important nonbonded interactions that are affected under these circumstances, and their count changed considerably. When the system reaches the equilibrate state, the hydrogen bond count attains its optimal number. Bringing equilibrated PZ, ZPI, and FXa proteins to each other in a ternary complex and running molecular dynamic simulation affect hydrogen bonds count. In a ternary complex of PZ-ZPI-FXa, there are three kinds of hydrogen bonds populations that may change during simulation: first is intramolecular hydrogen bonds count as H-bonds of PZ, ZPI, or FXa and second, intermolecular H-bonds as H-bonds between PZ and ZPI, between ZPI and FXa, and between PZ and FXa, and third is the hydrogen bond between proteins and solvent.

**Figure 8:**
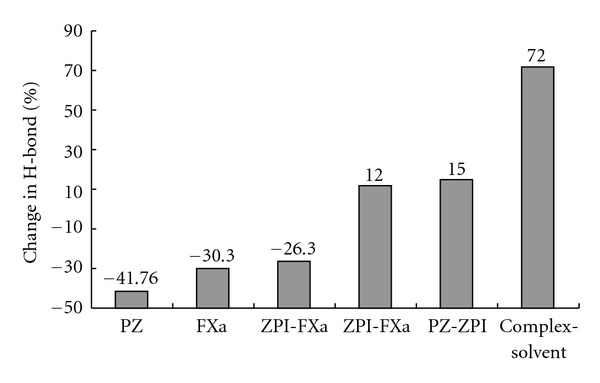
Changes in H-bonds counts calculated by subtracting the H-Bond counts of the first frame from last frame of trajectory file of ternary complex of PZ-ZPI-FXa and are expressed as percent of canges. Positive values indicate increased, and negative values indicate decreased count of H-bonds.

Figure 8 displays the changes in hydrogen bond count between the first and the last frame of trajectory files of simulated ternary complex. The main point in this figure is the increase in intermolecular hydrogen bonds count and increase in hydrogen bonds between ternary complex with surrounding solvent at the expense of the decrease in intramolecular hydrogen bonds.

## 4. Conclusion

Based on our findings and the previously postulated mechanism for PZ-mediated FXa inhibition by ZPI, we conclude that PZ, beside its action as plasma carrier for ZPI, by using its SP-like domain, binds to ZPI through forming noncovalent forces such as salt bridges and hydrogen bonds. Negative charges of GLA domain of PZ make strong ionic bonds with a positive charge of bivalent cation, calcium. The next positive charge of calcium ions at the vicinity of platelets membrane is used to bind GLA domain to negative charges of platelet membranes. These interactions cause PZ binding to the membrane probably where FXa has been previously immobilized. PZ binding to membrane induces conformational alterations including ZPI pulling down, RCL loop of ZPI insertion into FXa active site, and formation of active ternary complex of PZ-ZPI-FXa. The active complex leads to catalytically cleavage of ZPI at P1 site and enzyme inhibition [27, 30, 40].

## Figures and Tables

**Table 1:** The distance between alpha carbon of Arg255 and alpha carbons of three residues including Glu92, Glu320, and Arg319 are presented in angstrom. The distance between functional group of Arg255 and nearer functional groups of each of residues Glu92, Glu320, and Arg319 capable of forming hydrogen bonds is also included.

Residue	C*α*-C*α* distance (Å)		Hydrogen-binding groups distance (Å)	
	Before simulation	After simulation	Before simulation	After simulation
Arg255-Glu92	13.35	10.04	1.68	3.27
Arg255-Glu320	10.49	11.04	8.67	8.78
Arg255-Arg319	9.94	10.06	5.94	6.68
